# Medicinal value of sunflower pollen against bee pathogens

**DOI:** 10.1038/s41598-018-32681-y

**Published:** 2018-09-26

**Authors:** Jonathan J. Giacomini, Jessica Leslie, David R. Tarpy, Evan C. Palmer-Young, Rebecca E. Irwin, Lynn S. Adler

**Affiliations:** 1 North Carolina State University, Department of Applied Ecology, 100 Eugene Brooks Avenue, Raleigh, NC 27695 USA; 2 University of Massachusetts Amherst, Department of Biology, 611 North Pleasant Street, Amherst, MA 01003 USA; 3 North Carolina State University, Department of Entomology & Plant Pathology, Campus Box 7613, Raleigh, NC 27695 USA

## Abstract

Global declines in pollinators, including bees, can have major consequences for ecosystem services. Bees are dominant pollinators, making it imperative to mitigate declines. Pathogens are strongly implicated in the decline of native and honey bees. Diet affects bee immune responses, suggesting the potential for floral resources to provide natural resistance to pathogens. We discovered that sunflower (*Helianthus annuus*) pollen dramatically and consistently reduced a protozoan pathogen (*Crithidia bombi*) infection in bumble bees (*Bombus impatiens*) and also reduced a microsporidian pathogen (*Nosema ceranae*) of the European honey bee (*Apis mellifera*), indicating the potential for broad anti-parasitic effects. In a field survey, bumble bees from farms with more sunflower area had lower *Crithidia* infection rates. Given consistent effects of sunflower in reducing pathogens, planting sunflower in agroecosystems and native habitat may provide a simple solution to reduce disease and improve the health of economically and ecologically important pollinators.

## Introduction

Pollinators are critically important for the preservation of plant biodiversity, and provide billions of dollars in crop pollination annually^1,2^. Bees are the dominant pollinators of the majority of animal-pollinated flowering plants globally^3^ and are important for the production of many crops^4^. There have been mounting concerns about increased mortality in both honey bees and native bees^5^. Although a variety of factors are involved, pathogens have been strongly implicated in the decline of many bee species^5^. One of the most pressing concerns in the management of bee disease is the identification of factors that could reduce bee disease in natural and managed landscapes.

Many studies have examined the role of landscape factors, including plant diversity, on pollinator abundance and colony growth^6–9^, but the role of particular plant species in mediating bee-pathogen dynamics is largely unknown. For example, previous work has linked bumble bee pollen collection and colony growth to land-use patterns, and found that quantity, rather than quality, of pollen was most important for growth^8^. However, this work did not consider the role of pathogens. Conversely, a recent study that incorporated a range of landscape factors and pesticide use data found that use of the fungicide chlorothalonil was the best predictor of the pathogen *Nosema* in four declining bumble bee species^9^, but this work did not consider the role of particular plant species or pollen quality. Although pathogens can be horizontally transferred among bees at shared flowers^10,11^, and flower species can differ in their transmission probabilities^10,11^, there is currently no published work suggesting that particular plant species may play significant roles in mediating bee-pathogen dynamics.

Pollen is the sole source of lipids and protein for bees, and varies widely in nutritional content^12^, morphology, and chemistry^13^. Pollen nutritional quality, including protein, is important for individual bee size^14^ and metrics of colony performance^15,16^ and pollen macronutrient ratio shapes bumble bee foraging preferences^17^. Pollen quality also affects the expression of genes relating to host immune function^18^, and pollen starvation increases the likelihood of bees dying when infected with a common gut pathogen^19^. Previous work has shown that nectar chemistry can mediate bee disease^20^, and one study found that pollen from different plant species affects honey bee tolerance of the pathogen *Nosema ceranae* and expression of immune genes^21^. Thus, interspecific variation in pollen composition may have a critical but largely unknown effect not only on bee performance, but also interactions with pathogens^21–23^.

We conducted a series of laboratory experiments and a field survey to investigate the effect of pollen diet on bee disease and health using both the common eastern bumble bee, *Bombus impatiens* (Apidae), and the European honey bee, *Apis mellifera* (Apidae). Bumble bees can be infected with a diversity of pathogens, including *Crithidia bombi* (Trypanosomatidae), a protozoan gut pathogen contracted at flowers by fecal transmission^11^. *Crithidia* reduces learning and foraging efficiency in worker bees^24^, slows colony growth rates (especially at the start of the season^25^), increases worker mortality, and reduces queen fitness under stressful conditions^19^. *Crithidia* infection is common, with a prevalence of over 80% in *B. impatiens* in some regions^26^. Honey bees can also be infected by a diversity of pathogens, including an obligate intracellular pathogen *Nosema ceranae* (Microsporidia), which has been implicated in colony losses. Field experiments suggest that *Nosema* infection can cause a rapid collapse of otherwise healthy colonies^27^.

To test whether pollen from different plant species could influence bee-pathogen dynamics, we first compared the effects of pollen from three different plant species on bumble bee infection intensities. Upon finding that sunflower pollen dramatically reduced infection intensity in multiple experiments, we assessed the effects of sunflower pollen on bumble bee microcolony performance in the presence and absence of infection. We then explored the generality of our findings with another bee-pathogen system, the European honey bee and the pathogen *Nosema*. Finally, we tested the hypothesis that increased sunflower crop area reduces *Crithidia* in wild bumble bees at the farm level, to assess whether our laboratory results could extend to the field. Taken together, our findings suggest that sunflower pollen may serve as a novel tool to manage bee disease dynamics.

## Results

### Effects of pollen diet on *Crithidia* in bumble bees

**Figure 1:**
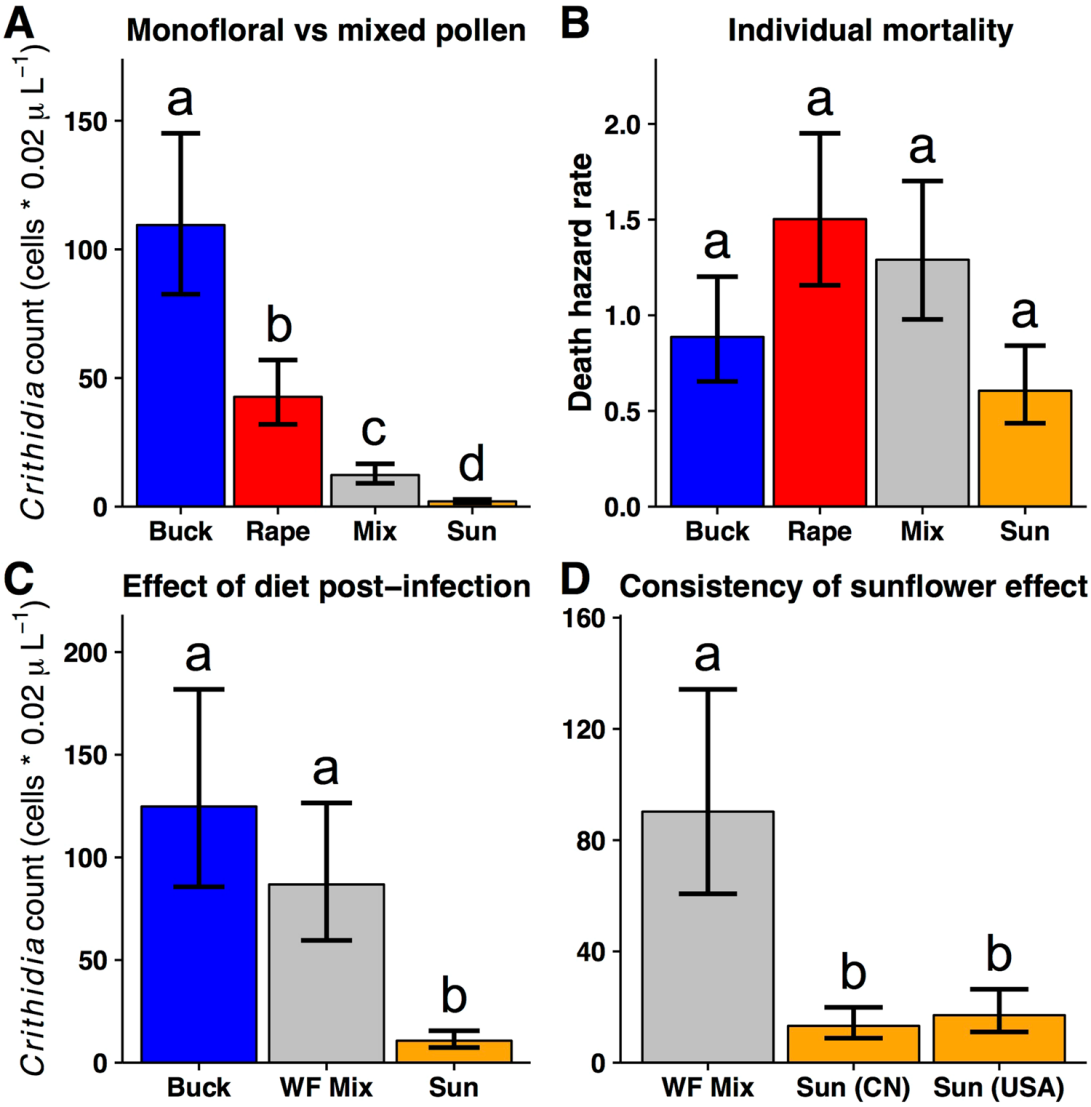
(**A**) Effects of pollen diets on *Crithidia* infection in individual *Bombus impatiens* workers. Bees were inoculated with *Crithidia* and fed a monofloral pollen diet commonly grown in large monocultures in agroecosystems: sunflower (*Helianthus annuus*; Sun), buckwheat (*Fagopyrum cymosum*; Buck), rapeseed (*Brassica campestris*; Rape), or a mixed diet composed of equal weights of the three monofloral pollens (Mix). (**B**) Pollen diets did not significantly affect rate of worker death over the 7 d experiment shown in (**A**). Y-axis shows exponentiated hazard rates ±1 standard error. (**C**) *Crithidia* infection was allowed to build for one week post-inoculation before providing pollen treatments: sunflower (Sun), buckwheat (Buck), or a wildflower pollen mixture (WF Mix). (**D**) Inoculated bees were fed sunflower pollen from two sources, China (CN) or USA (USA), or a control wildflower pollen mixture (WF Mix). Bars and error bars indicate negative binomial model means ±1 standard error back-transformed (i.e., exponentiated) from the scale of the linear predictor. *Crithidia* counts represent raw counts of cells diluted in a gut homogenate. Error bars represent uncertainty in fixed effects portions of models only, and do not account for variability due to random effects. Different letters above each bar within panels indicate significant differences based on Tukey’s HSD tests.

We first tested the hypothesis that pollen from different plant species varies in its effects on bumble bee infection intensities. We focused on three monofloral pollens commonly grown in large monocultures in agroecosystems and visited by bees: rape (*Brassica campestris*), sunflower (*Helianthus annuus*), and buckwheat (*Fagopyrum cymosum*), as well as a mixed diet composed of the three monofloral pollens. We experimentally inoculated bees with *Crithidia*^20^, provided them with monofloral pollen diets of each species or the pollen diet mix, and measured subsequent infection intensity. Sunflower pollen significantly reduced *Crithidia* infection in bumble bees compared to all other pollen diets (χ^2^_(3)_ = 111.2, P < 0.001). Infection levels were 20- to 50-fold lower in bees fed sunflower pollen than either rape or buckwheat pollen, respectively (Fig. 1A). Moreover, two-thirds of the sunflower-fed bees had no detectable infection after one week of treatment. We found no effect of pollen diet on bee survival (χ^2^_(3)_ = 4.04, P = 0.257; Figs 1B; S1; Supplementary Information: Text 1), suggesting minimal mortality costs. In a separate experiment, we allowed infection levels to build for one week before providing pollen treatments, and we found a 5- to 8-fold reduction of infection within bees fed sunflower pollen compared to a wildflower pollen mixture or buckwheat pollen, respectively (χ^2^_(2)_ = 17.2, P < 0.001; Fig. 1C).

The medicinal effects of sunflower pollen were consistent across pathogens collected from two locations, as well as with two different sources of sunflower pollen. In addition to our original results using *Crithidia* from Massachusetts, USA (Fig. 1A), sunflower pollen also reduced *Crithidia* infection intensity by 30-fold in a separate experiment where bees were infected with a pathogen lineage isolated in North Carolina, USA (Pollen treatment χ^2^_(1)_ = 30.7, P < 0.001; Fig. S2). We also compared domesticated sunflower pollen from two sources (Methods). Both showed medicinal effects compared to bees fed a wildflower pollen mixture (Pollen treatment: χ^2^_(2)_ = 23.6, P < 0.001; Fig. 1D), with sunflower pollen reducing disease at least 4-fold. There was no difference in pathogen reduction between the two sunflower sources (Z = 0.601, P = 0.82). Furthermore, the medicinal effects of sunflower pollen were not associated with any potential pesticide residues in the pollen diets (Supplementary Table 1).

### Costs and benefits of sunflower pollen on bee health, reproduction and *Crithidia*

**Figure 2:**
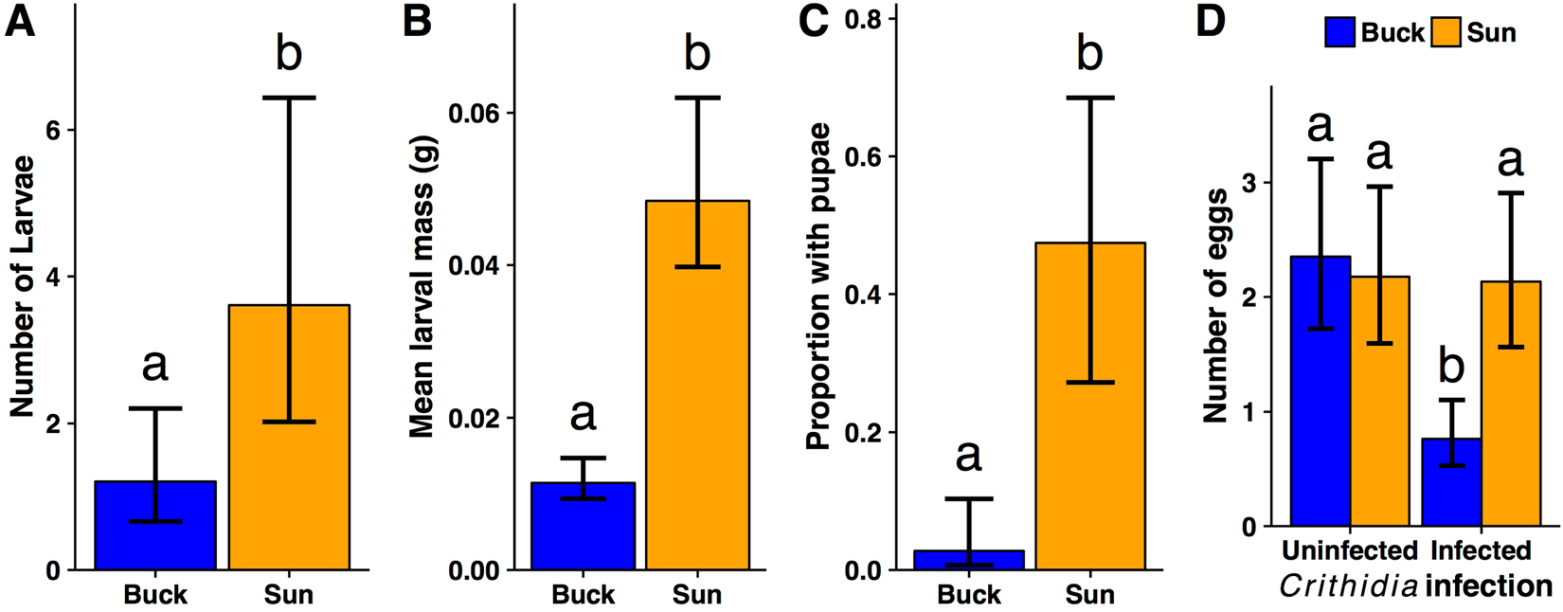
*Bombus impatiens* microcolony performance. Microcolonies were fed either buckwheat (Buck) or sunflower (Sun) pollen diets and either inoculated with *Crithidia* (Infected) or a *Crithidia*-free control solution (Uninfected). Infection did not significantly affect responses in A-C and so responses were averaged across infection treatments for these panels. (**A**) Mean number of larvae produced, (**B**) mean total larval mass, (**C**) proportion of microcolonies that produced pupae during the experiment, and (**D**) mean number of eggs produced. *Crithidia* infection reduced egg production in microcolonies fed buckwheat pollen, but not sunflower pollen. For all panels, error bars indicate ±1 standard error. Error bars represent uncertainty in fixed effects portions of models only, and do not account for variability due to random effect.

To ask how sunflower pollen affects bee performance, we conducted a factorial experiment using microcolonies of queenless workers with infection (yes or no) crossed by pollen diet treatment (sunflower or buckwheat). We used buckwheat as a comparison to sunflower pollen because it supported high *Crithidia* levels (Fig. 1A) but has a similar protein content as sunflower^28^, allowing us to compare pollens of relatively similar protein content but different effects on *Crithidia*. Over the course of the experiment, bees consumed more sunflower than buckwheat pollen (χ^2^_(1)_ = 66.67, P < 0.001; Fig. S3; Supplementary Information: Text 2), suggesting that the medicinal benefits of sunflower pollen were not due to lower pollen consumption, which can independently reduce *Crithidia* infection^29^. Consumption of sunflower pollen significantly increased nearly every measure of microcolony reproduction compared to buckwheat pollen, including number of eggs, larval number and mass, and probability of producing pupae (P < 0.004 in all cases; Fig. 2), but was marginally associated with increased worker mortality (χ^2^_(1)_ = 3.78, P = 0.051; Fig. S4). Moreover, infection reduced egg production in bees fed buckwheat but not sunflower (Infection x Pollen interaction χ^2^_(1)_ = 10.34, P = 0.0013; Fig. 2D), indicating that for this performance metric, sunflower pollen consumption can alleviate the negative effects of infection.

### Effects of pollen diet on *Nosema* in honey bees

**Figure 3:**
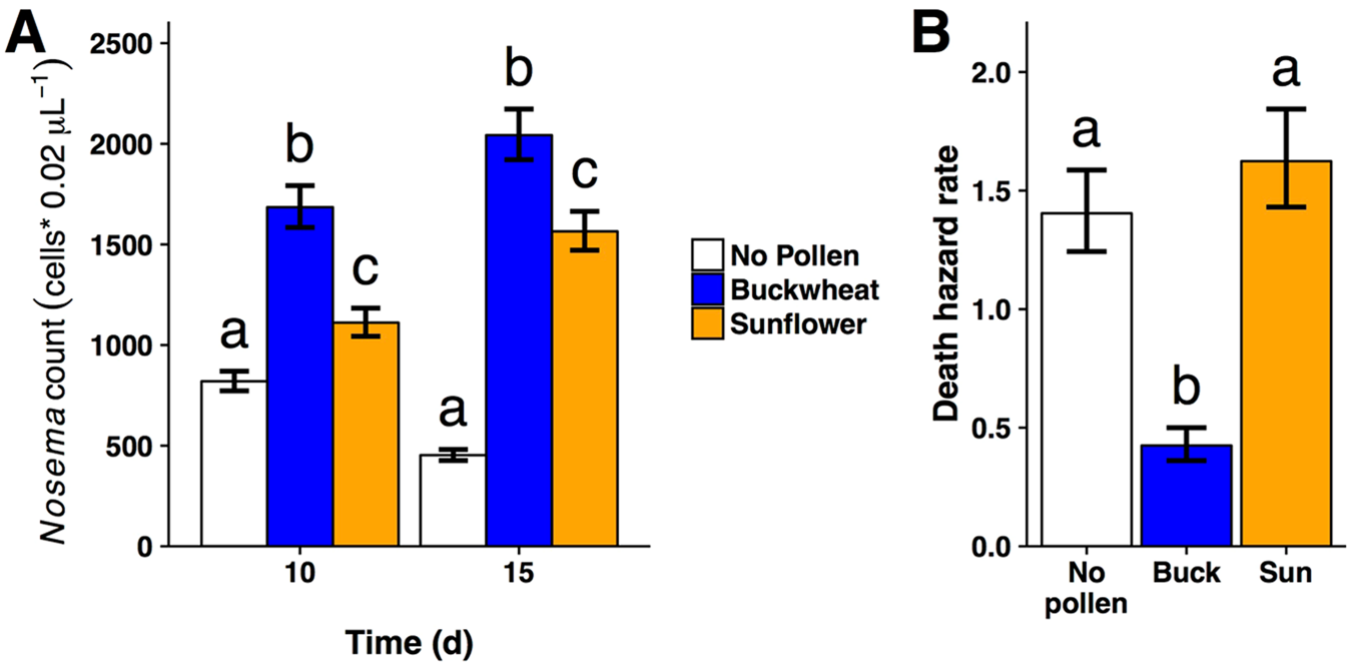
Effects of pollen diet on *Nosema* infection in honey bees (*Apis mellifera*). (**A**) Sunflower pollen reduced *Nosema* infection in honey bees by an average of 29% compared to buckwheat pollen across the two time periods. Bars and error bars indicate negative binomial model means ±1 standard error back-transformed (i.e., exponentiated) from the scale of the linear predictor. Error bars represent uncertainty in fixed effects portions of models only, and do not account for variability due to random effect. (**B**) Exponentiated hazard rates ±1 standard error for mortality on different pollen diets. Sunflower-fed bees died at nearly four times the rate of buckwheat-fed bees and had equivalent survival to bees with no pollen. Lower-case letters indicate significant differences based on post hoc pairwise comparisons; in (**A**), comparisons are made within each time point (10 d and 15 d).

Having shown strong, consistent reductions in infection in bumble bees fed sunflower pollen, we then tested the effect of sunflower pollen on the pathogen *Nosema* in European honey bees. We experimentally infected groups of honey bees with *Nosema* and then fed them either buckwheat pollen, sunflower pollen, or no pollen as a negative control. At both 10 d (Z = −4.72, P < 0.001) and 15 d (Z = −3.06, P = 0.006) post-infection, sunflower pollen reduced *Nosema* infection in honey bees relative to buckwheat pollen (Fig. 3A; Supplementary Information: Text 3). Averaged across both time periods, infection intensity was 29% lower in sunflower- than buckwheat-fed bees, although still more than twice as high as in bees denied pollen. Despite this reduction in infection, the consumption of sunflower pollen came at a cost of increased mortality relative to buckwheat-fed bees (hazard ratio = 3.8, Z = 5.175, P < 0.001) and was similar to mortality in bees given no pollen (Z = −0.75, P = 0.74; Figs 3B and S5).

### Effect of sunflower plantings on *Crithidia* in bumble bees at the farm scale

**Figure 4:**
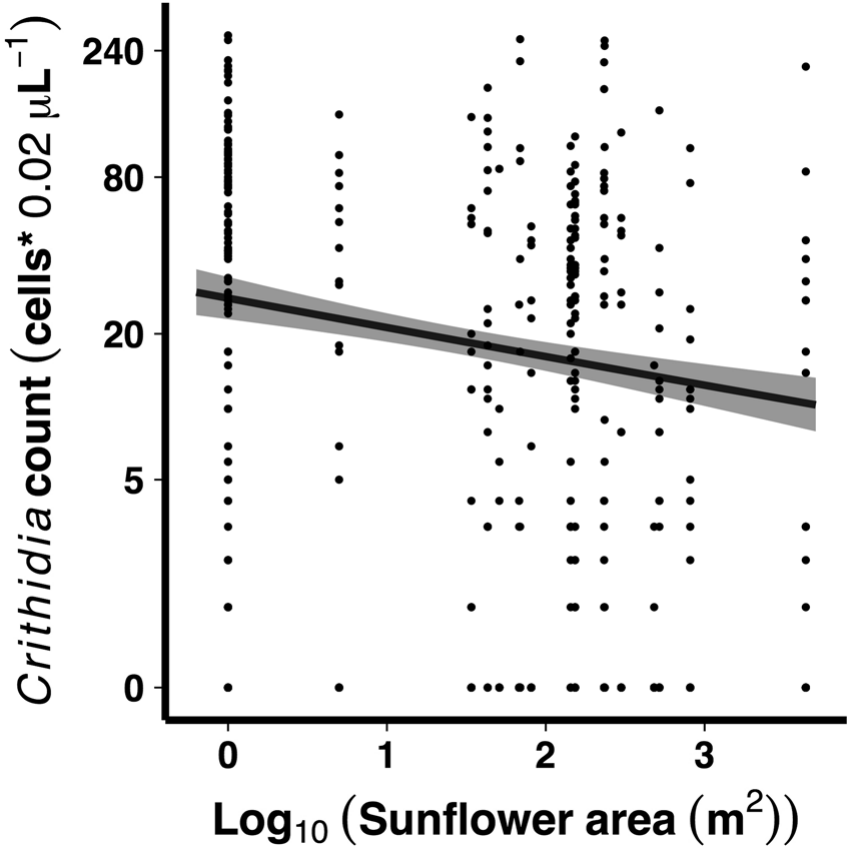
Negative relationship between the area of sunflower planted on farms and *Crithidia* infection intensity in *Bombus impatiens* workers. Line and shaded band indicate back-transformed mean *Crithidia* counts for area of sunflower planted ±1 standard error; points show counts for individual bees.

We sampled worker *B. impatiens* from 22 farms (approx. mean distance of 2.5 km between farms) in western Massachusetts, USA, between July 27 and September 18, 2015. Farms ranged in size between 0.3 ha and 62.9 ha, with an average size of 16.3 ± 3.9 ha (Mean ± SE). We found a significant, negative relationship between the area of sunflower planted on farms and *Crithidia* infection intensity (linear mixed model β = −0.26 ± 0.010 SE, likelihood ratio χ^2^_(1)_ = 6.88, P = 0.009; Fig. 4). This corresponds to a 23.2% decrease in infection intensity on the linear scale (95% CI: 6.25% to 37.0%) for every 10-fold increase in sunflower area, or a 50% decrease for every 425-fold increase in sunflower area. Sampling date and sunflower area were not confounded (Pearson’s r = 0.02). However, there was a significant decrease in infection intensity over the course of the sampling period, which spanned 52 d from the beginning of August through late September (β = −0.038 ± 0.013 SE, likelihood ratio χ^2^_(1)_ = 14.46, P < 0.001). We sampled farms with different management practices (organic and conventional) and different varieties of sunflower (see Methods), but farm did not explain significant variation in infection intensity (χ^2^_(1)_ = 0.24, P = 0.62).

## Discussion

Sunflower pollen dramatically and consistently reduced *Crithidia* in bumble bees across a series of laboratory experiments. Sunflower pollen also resulted in greater bumble bee microcolony reproduction than buckwheat pollen, which matched sunflower pollen in protein content but did not reduce *Crithidia* infection. Additionally, these results were reflected in a field survey of pathogen infection intensity, which was reduced on farms with greater sunflower area. Sunflower is a common native plant in much of the US and is widely planted for agriculture worldwide. Thus, the consequences of this bee-pathogen-sunflower interaction may be widespread; in the US, almost two million acres are planted with sunflower^30^, and in Europe, about ten million acres are planted with sunflower annually^31^. Furthermore, we found a significant relationship between infection intensity and sunflower coverage without considering other factors that could also explain variation in parasite infection, such as farm management practices, farm size, other flowering crops, or landscape context. Thus, while there is substantial unexplained variation in *Crithidia* infection intensity (Fig. 4), our results suggest that the relationship between infection and sunflower plantings is consistent across a wide range of contexts.

We also found that sunflower pollen reduced *Nosema* in honey bees, although the effect was less dramatic than it was for *Crithidia* in bumble bees. Our results are consistent with previous work that demonstrates *in vitro* antimicrobial effects of secondary metabolite extracts from sunflower pollen against bacteria and fungi^32^ and the medicinal value of sunflower honey against *Nosema* in honey bees^33^, but vastly expands the breadth and medicinal potential of sunflower pollen by demonstrating dramatic reductions in a distantly related pathogen of bumble bees. However, despite the reduction in *Nosema* infection, the consumption of sunflower pollen by honey bees came with a cost of greater mortality. Thus, any anti-parasitic benefits of sunflower pollen need to be viewed within the context of mortality costs for honey bees. Future work that examines the relative benefits of sunflower pollen consumption across a variety of doses for healthy vs. infected bees may indicate appropriate procedures for use as commercial dietary supplements.

Although our experiments were not designed to determine the mechanism(s) behind the medicinal effects of sunflower, the results allow us to rule out some potential mechanisms and suggest others for future research. The nutritional components of sunflower, buckwheat, rape, and many other species of pollen have been explored^12,15,28,34,35^, and a low protein pollen diet may induce physiological costs^15,36^ and reduce longevity of parasitized bees^21^. Although honey bee-collected sunflower pollen is notably low in total protein compared to pure pollen collected directly from flowers^28,35^, buckwheat is equally low^28^ (both 14–15 g/100 g compared to 27 g/100 g in rape), suggesting that low protein is not the mechanism. Similarly, although sunflower pollen is low in some amino acids including methionine, glutamic acid and proline^28,35^, all of these components are also relatively low in buckwheat pollen and much higher in rape pollen^28^, which does not correspond with relative medicinal effects. By contrast, concentrations of some key fatty acids correspond with relative medicinal quality (i.e., highest in sunflower, intermediate in rape, and low in buckwheat), including linoleic, decanoic and lauric acids, which are antimicrobial in honey bee brood combs^37^, and must be acquired in the diet^38^. Because fatty acids are ubiquitous components of pollen^37^, identifying fatty acids that affect bee disease could have broad implications for discovering additional medicinal pollens, as well as breeding medicinal traits.

There are four additional, non-mutually exclusive hypotheses that could explain the medicinal properties of sunflower pollen. First, Asteraceae pollen is notable for its conspicuous spines on the outer coat^39^. Given that *Crithidia* is a gut parasite that attaches to the hindgut wall^40^, sunflower pollen could reduce parasitism by scouring the hindgut of parasite cells. Second, if sunflower pollen has laxative properties, it may decrease gut passage time and flush *Crithidia* and *Nosema* from bumble and honey bees before the pathogens can adhere to the gut. In a similar vein, the nectar alkaloids nicotine and anabasine can reduce *Crithidia* infection^20^, and these compounds also reduced gut passage time and sugar assimilation in the Palestine sunbird^41^. Although gut physiology is certainly different between sunbirds and bees, these results are consistent with the hypothesis that diet components could act as laxatives. Third, sunflower pollen could affect insect immune function. Recent work showed that sunflower pollen extract increased rather than suppressed *Crithidia* growth *in vitro*^42^, suggesting that the effect of sunflower pollen on *Crithidia* may be mediated by the bee host environment. Insect immune function occurs through a variety of processes, including melanization and encapsulation of foreign material, which is initiated by the activation of phenoloxidases^43^. It is possible that sunflower pollen may mediate *Crithidia* infection through changes in PO activity, encapsulation, or fat body production. Fourth, indirect pathogen resistance may also be mediated through changes in the host microbiome rather than the host itself. Gut microbiota can play a key role in *Bombus* resistance to *Crithidia*^44,45^, and diet can alter bee microbiome communities^46^. Thus, sunflower pollen may increase bee resistance to *Crithidia* via changes in the gut microbiome. Further research is needed to address each potential mechanism.

In nature, pollen consumption by bees will be affected not only by availability, but also by bee preference. For example, bumble bees prefer to visit plant species that produce pollen with a high protein to lipid ratio^17^. However, bees can also alter foraging preferences when infected by *Crithidia*^47,48^. Interestingly, infection with *Nosema* increased honey bee attraction to sunflower honey, which also reduced infection^33^, suggesting self-medication behavior. However, honey bees have also demonstrated a relatively low attraction to sunflowers, preferring to forage on other plants surrounding sunflower fields, including corn, clover and flowering trees^49,50^. Controlled experiments that assess bee preference for sunflower as a function of pathogen infection will yield important ecological insights. In addition to foraging preference, farm management practices can also shape bee disease dynamics. For example, a greater use of the fungicide cholorothalonil was positively related to *Nosema* prevalence in four declining North American bumble bee species^9^. Interestingly, we found that the negative relationship between infection and sunflower crop area was robust to farm management practices (organic vs. conventional). Nonetheless, understanding bee disease dynamics at the landscape level will require knowledge of the combined effects of flower species identity, bee foraging preferences, and interactions with farm management practices.

For both food security and biodiversity conservation, there is a critical need to move beyond documentation of pollinator declines and identify solutions to reduce bee disease and improve bee health. Sunflower pollen reduced the severity of infection by pathogens in bee species that are important for pollination services in natural and agroecosystems. Many beekeepers already provide pollen supplements to their colonies, and all levels of government, as well as growers, nonprofits, and the general public, are investing in plantings to improve pollinator habitat^51^. As both a domesticated crop and native wild species, sunflower could be prioritized for inclusion in agroecosystems and regionally appropriate native habitat. Our discovery that sunflower pollen reduced infection of multiple bee pathogens suggests the potential for simple, easily implemented approaches that could be tested for their ability to reduce disease and increase bee health.

## Methods

### Effects of pollen diet on *Crithidia* in bumble bees

#### *Crithidia* inoculum

Infected (‘source’) colonies were used to make *Crithidia* inoculum. The original *Crithidia* cells infecting colonies came from three wild *B. impatiens* workers collected from Stone Soup Farm (Hadley, MA, USA: 42.363911 N, −72.567747 W) unless otherwise noted. To make inoculum, bees were dissected from the source colony daily using an established protocol^20^. Bee digestive tracts (excluding the honey crop) were removed, placed into a 1.5 mL microcentrifuge tube with 300 μL of 25% strength Ringer’s solution (Sigma-Aldrich, St. Louis, MO, USA), finely ground, and vortexed for 5 seconds. Each sample was allowed to rest at room temperature for 4–5 hours. *Crithidia* cells were counted from a 0.02 μL sample per bee with a Neubauer hemacytometer^20^. We mixed 150 μL of the supernatant with 25% strength Ringer’s solution to achieve a concentration of 1200 cells μL^−1^. The inoculum was then mixed with an equal volume of 50% sucrose solution to yield inoculum with 600 cells μL^−1^ and 25% sucrose. Experimentally infected bees were starved for 4–6 hours and then fed a 10 μL drop of inoculum with 6,000 *Crithidia* cells, which is within the range of concentrations bees are exposed to when foraging on flowers in the wild^52^. Only bees that consumed the entire droplet were used in experiments.

#### Monofloral and mixed pollen

Monofloral pollen diets (rape, sunflower or buckwheat – *Brassica campestris*, *Helianthus annuus* and *Fagopyrum cymosum*, respectively) were obtained by sorting honey bee collected pollen pellets (Changge Hauding Wax Industry, China) initially by color. We then verified microscopically that pollen pellets within treatment were morphologically consistent and as expected for that species. Pollen was provided to bees as a paste made by mixing ground pollen pellets with distilled water to achieve a uniform consistency, which required different amounts of water depending on pollen species (pollen: water ratio: sunflower & buckwheat: 5:1; rape: 1.67:1; pollen mix of equal weights of the three monofloral pollens: 3.33:1).

Newly emerged adult worker bees (callows) obtained from pupal clumps were removed from six uninfected *B. impatiens* colonies (n = 272 bees). All *B. impatiens* colonies were provided by BioBest LTD (Leamington, Ontario, Canada), and experimental colonies were confirmed to be pathogen-free bi-weekly by screening five workers (see *Crithidia* inoculum). We regularly supplied all colonies with pollen loaves made of 30% sucrose solution mixed with ground honeybee–collected wildflower pollen (Koppert Biological Systems; Howell, MI, USA). Each day, newly emerged callows were collected from pupal containers, weighed to the nearest 0.01 mg, and randomly assigned to one of the four pollen diets. Bees were randomly assigned to treatment within experimental colony and, when relevant, date of emergence, for all experiments here and below. Bees were housed individually in a growth chamber in darkness at 28 °C and fed 500 μL of 30% sucrose solution and a small ball of their respective pollen treatment daily for 9 days. Bees were inoculated two days after emergence, so that bees consumed their respective pollen treatments both before and after infection.

*Crithidia* infection intensity was measured as *Crithidia* cells per 0.02 μL (hereafter “cell counts”) one week after bees were infected (n = 234 bees due to mortality). After 7 d, *Crithidia* infection intensity reaches a sufficient level for measurement within the bee host^53^. Each experimental bee was dissected (see *Crithidia* inoculum). We removed the right forewing of each bee and mounted them on glass slides to measure radial cell length, a proxy for bee size^54^.

#### Consistency with a different pathogen strain

*Crithidia* infection can be heavily influenced by genotypic variation in hosts and pathogens^55^, which may yield genetically distinct strains with varying susceptibility to host immune defenses^56^ and potentially responses to pollen diet. Thus, we repeated our experiment testing the effects of pollen diet on a different set of colonies infected with a strain obtained from wild *B*. *impatiens* collected in Raleigh, North Carolina, USA (J.C. Roulston Arboretum: 35.794056 N, −78.698186 W). Given the strong negative effects of sunflower pollen on *Crithidia* (see Fig. 1A), we used only sunflower pollen (*H. annuus*) and buckwheat pollen as our control (*F. cymosum*). In addition, adult workers (rather than newly emerged callow bees) were used in this experiment to ensure that results were consistent across bees of varying ages. Worker bees were used from three colonies, and bees were inoculated and *Crithidia* pathogen loads were measured (n = 149 bees).

#### Effect of diet post-infection

We tested whether sunflower pollen could reduce *Crithidia* infection in bees that already reached sufficient infection levels. Individual *B. impatiens* adult workers from three colonies were inoculated with *Crithidia* (North Carolina, USA strain) and fed a wildflower pollen mixture (Koppert Biological Systems; Howell, MI, USA) and 30% sucrose solution for 7 days. Each bee was then randomly assigned to one of three pollen diets: sunflower, buckwheat, or the same wildflower mix for 7 more days. By including a wildflower mix pollen treatment, we were able to compare monofloral pollen treatments to a more natural and diverse mix of pollens. Bees were then sacrificed (n = 74) and *Crithidia* pathogen loads were measured.

#### Consistency using two sources of sunflower pollen

Domesticated sunflower is a major oil crop distributed worldwide^35^. Breeding practices have modified a wide array of economically important traits, including seed and oil production^57^, resistance to plant diseases and pests^58^, and resistance to drought^59^. We compared the medicinal effects of sunflower pollen from China versus sunflower pollen from the USA. Adult *B*. *impatiens* workers from three colonies were inoculated with *Crithidia* (n = 120 bees) and fed either sunflower pollen collected from an organic farm in Wisconsin, USA (44.731641 N, −91.948666 W, Cobalt II cultivar - NuSeed Inc.), sunflower pollen collected in China (Changge Hauding Wax Industry, China), or the wildflower pollen mixture. We measured pathogen loads (n = 110 bees) after 7 days.

#### Statistical analyses

All statistical analyses here and below were conducted using R version 3.1.2^60^ (Supplementary Information: Methods 1). To test how pollen diets affected *Crithidia* infection intensity, generalized linear mixed models were used to analyze *Crithidia* cell counts using “glmmTMB”^61^, with pollen diet as a fixed effect, bee size as a covariate, and experimental bee colony and inoculation date (if applicable) as random effects. Significance of terms was evaluated with a likelihood ratio chi-squared test, implemented via the “drop1()” function. Tukey’s HSD tests were used for post hoc pairwise comparisons. All bees that died before their scheduled dissection date were excluded from analyses. To test how pollen diets affected bee survival, mixed-model Cox proportional hazards tests were used^62^, with pollen diet and bee size as fixed effects, and inoculation date (if applicable) and experimental bee colony as random effects. To assess the effects of pollen diet on mortality, log-likelihood of models were compared with and without pollen diet treatment as a predictor. Significance of terms was tested with a Wald chi-squared test, implemented via the Anova function in package “car”^63^. Plots (here and throughout) were produced with ggplot2^64^, survminer^65^ and cowplot^66^.

### Costs and benefits of sunflower pollen for bee health, reproduction and *Crithidia*

Using queenless *B*. *impatiens* microcolonies, we tested the impact of pollen diet and *Crithidia* infection on mortality, reproduction and *Crithidia* infection in a 2 × 2 factorial design manipulating pollen diet (sunflower or buckwheat) and *Crithidia* infection (uninfected or infected). When unmated workers are isolated from the queen, one will gain dominance and lay haploid (male) eggs. Microcolonies are an effective approach to estimate the effects of diet and pathogen infection on whole-colony reproduction^15,20,67^. We used 20 replicate microcolonies per treatment for a total of 80 microcolonies, carried out in two rounds (or blocks) of 40 microcolonies, with five workers per microcolony. The first 40 microcolonies were constructed using workers from two colonies, with 5 replicates per treatment per colony of origin. The second set of 40 microcolonies were constructed from two new colonies of origin.

Microcolonies were randomly assigned to infection and diet treatments within rounds and colonies of origin. Bees were inoculated with *Crithidia* as in ‘*Crithidia* inoculum’ or given a sham control inoculum of 10 µL of sucrose solution without *Crithidia* cells. We maintained microcolonies in a growth chamber at 28 °C in darkness and fed them 400 mg of pollen each and ad libitum 30% sucrose solution, replaced and replenished 5 d per week. Pollen diets were made as in ‘Monofloral and mixed pollen’. We measured pollen and sucrose solution consumption (in g) 5 days per week, calculating the total mass consumed (or used) per bee per hour. Pollen consumption was corrected for evaporation by subtracting the average weight lost to evaporation over 24 hr for each pollen type. To determine the average weight lost to evaporation, 15 samples of each pollen type were placed into empty microcolony containers without bees and in the same growth chamber for 24 hr. Each pollen sample was weighed at 0 hr and at 24 hr to determine the net weight lost to evaporation.

For each microcolony, we recorded the date of first eggs laid, male emergence and weight (which occurred in 5 of the 80 microcolonies) and worker mortality. Microcolonies were terminated 35 days post-egg laying, or if 4 out of the 5 worker bees died. We then measured *Crithidia* infection in the remaining worker bees (see *Crithidia* inoculum) and bee size. For each microcolony, the number of eggs, larvae, and pupae produced was counted and weighed. Because bees within microcolonies can vary in size and social dominance, which can affect food consumption and microcolony reproduction, we calculated a metric of within-microcolony size dimorphism [(largest bee radial cell/smallest bee radial cell) − 1]^20,68,69^ for use as a covariate.

To analyze pollen and nectar consumption, *Crithidia* infection intensity, and microcolony reproduction, generalized linear mixed effects models were fit with distributions specific to the type of data analyzed (Supplementary Information: Methods 2). Unless otherwise noted, all models included fixed effects of pollen diet (sunflower or buckwheat), infection treatment (infected or uninfected), and (when significant) their interaction. All statistical tests included block as a random effect, which corresponded to microcolonies inoculated on the same day. The block effect accounted for variation due to colony of origin (because each inoculation day used a different colony of origin) and variation due to different inoculation dates.

### Effects of pollen diet on *Nosema* in honey bees

Newly emerged worker honey bees from three colonies were mixed together and placed into cages in groups of 50 bees per cage^70^ with 50% sucrose solution. We experimentally infected the bees in each cage using a *Nosema* spore sucrose solution with a concentration of approx. 333,333 spores per bee^71,72^. Cages were randomly assigned to a pollen diet treatment and given a single 20 g ball of sunflower or buckwheat pollen paste, or no pollen as a negative control for 15 days. Prior studies have shown that *Nosema*-infected honey bees that do not consume pollen have significantly lower *Nosema* infection intensity than bees provided with pollen^71^. There were 11–12 replicate cages per pollen diet treatment. On days 10 and 15, samples of five bees and 10 bees per cage, respectively, were sacrificed to quantify *Nosema* infection intensity^71,72^. Any bees that died during the experiment were counted and removed from their cages.

We used generalized linear mixed effects models (R package glmmTMB) to test whether pollen diet affected *Nosema* infection intensity (spores per mL) on days 10 and 15 (Supplementary Information: Methods 3). *Nosema* infection intensity was used as the response variable; pollen treatment, days since inoculation, and their interaction were used as fixed predictors; cage was included as a random effect to account for repeated measures on each cage. Differences in survival were tested using a Cox Proportional Hazards mixed-effects model fit using “coxme”^62^, with pollen diet as a fixed effect and cage as a random effect.

### Effect of sunflower plantings on *Crithidia* infection in bumble bees at the farm scale

Bees were collected directly from sunflowers if available, or else from a variety of flowering crops. Each farm was sampled on a single date. We quantified the area of sunflower grown at each farm in m^2^. We sampled a total of 667 *B*. *impatiens* workers (range: 19–62 bees per farm); all bees were sacrificed and we measured *Crithidia* infection (as in *Crithidia* inoculum).

We tested for spatial autocorrelation using a Monte-Carlo Mantel test and a Moran’s I test using the “ape” and “ade4” packages in R^73,74^. We found no indication of spatial autocorrelation (P > 0.15), and so considered farms to be independent sampling locations. We analyzed infection intensity (*Crithidia* cell counts) with a generalized linear mixed model with negative binomial error distribution using the “glmmTMB” package in R^61^. Sunflower area (log_10_ area (m^2^)) and Julian date of sampling were used as fixed covariates; farm was included as a random effect to account for non-independence of bees within a farm. Sampling date and sunflower area were not confounded (Pearson’s r = 0.02). Significance of predictors was tested by likelihood ratio chi-squared tests, implemented via the “drop1” function in R.

### Pesticide analysis

To ensure that results were not associated with pesticide residues on pollen, the USA and Chinese sunflower, buckwheat, and the wildflower mix pollens were analyzed for 213 pesticides and other agrochemicals (Agricultural Marketing Services’ National Science Laboratories, United States Department of Agriculture, Gastonia, NC USA) (Supplementary Information: Table 1).

## Electronic supplementary material

Supplementary Information

## Data Availability

All data and custom scripts used for statistical analysis generated from this project is avaliable here: https://github.com/FatherofEverest/Medicinal-value-of-sunflower-pollen-against-bee-pathogens-Data-Availability.
