# Supplementary material for: Medicinal value of sunflower pollen against bee pathogens
Source: Sci Rep. 2018 Sep 26;8:14394. doi: 10.1038/s41598-018-32681-y (PMC6158195; doi:10.1038/s41598-018-32681-y)
Supplement: Supplementary file 1 — Supplementary Information [file 41598_2018_32681_MOESM1_ESM.docx]

**Supplementary Information**

**Title:**  Medicinal value of sunflower pollen against bee pathogens

**Authors:** Jonathan J. Giacomini^1*^, Jessica Leslie^2^, David R. Tarpy^3^, Evan C. Palmer-Young^2^, Rebecca E. Irwin^1^†, and Lynn S. Adler^2^†

**Affiliations:**

^1^ North Carolina State University, Dept. of Applied Ecology, 100 Eugene Brooks Avenue, Raleigh, NC 27695, USA

^2^ University of Massachusetts Amherst, Dept. of Biology, 611 North Pleasant Street, Amherst, MA 01003, USA

^3^ North Carolina State University, Department of Entomology & Plant Pathology, Campus Box 7613, Raleigh, NC 27695, USA

*Corresponding author: Jonathan Giacomini, email: jjgiacom@ncsu.edu, ORCID ID: 0000-0002-0151-894X

† equally contributing senior authors

**Supplementary Text:**

**1. Effects of pollen diet on *Crithidia* in bumble bees.** In all experiments, *Crithidia* populations grew rapidly in infected hosts after inoculation, reaching up to 5,777 cells μL^-1^ in bees in wildflower control and buckwheat pollen diet treatments.

*Monofloral and mixed pollen.* Post-hoc analysis revealed all pairwise combinations of pollen diet had statistically different effects on *Crithidia* infection intensity (Tukey’s HSD, *P*<0.05 in all comparisons; Fig. 1A). Additionally, almost two-thirds of the bees fed sunflower pollen and one-third of the bees fed the three-pollen polyfloral mix had no detectable *Crithidia* infection; fewer than 10% of bees fed canola and buckwheat pollen had no detectable infection. There was no significant difference in mortality for the different pollen diets (Fig. 1B, Fig. S1). The covariate bee size was significantly negatively related to *Crithidia* infection intensity (*χ^2^*_(1)_ = 6.64, *P* = 0.010).

*Effect of diet post-infection.* Post-hoc analysis revealed significant pairwise differences in the effect of sunflower pollen compared to wildflower and buckwheat pollen (*Z >* 3.95, *P* < 0.05 in both cases), but not between wildflower and buckwheat pollen (*Z* = 0.68, *P* = 0.77; Fig. 1C).

*Consistency with a different pathogen strain*. Sunflower pollen diet reduced *Crithidia* collected in North Carolina, USA compared to buckwheat pollen diet (*χ^2^*_(1)_ = 30.7, *P* < 0.001), with sunflower reducing infection 30-fold (Fig. S2). The covariate bee size was significantly positively associated with *Crithidia* infection intensity (*χ^2^*_(1)_ = 10.17, *P* = 0.001).

*Consistency using two sources of sunflower pollen*. Post-hoc analysis revealed a significant difference between the Chinese sunflower pollen diet and the wildflower pollen mix (*Z* = 4.909, *P* < 0.001), as well as a significant difference between the US sunflower pollen and the wildflower pollen mix (*Z* = 3.95, *P* < 0.001). There was no difference between the US and Chinese sunflower pollen diets in terms of reduction in *Crithidia* infection intensity (*Z* = 0.60, *P* = 0.82; Fig. 1D).

**2. Costs and benefits of sunflower pollen on bee health, reproduction and *Crithidia*.** *Crithidia* populations again grew in uninfected hosts receiving buckwheat pollen diet; counts of buckwheat-fed bees averaged 2000 cells µL^-1^ in diluted gut homogenate after the termination of microcolonies. Sunflower pollen significantly reduced *Crithidia* infection intensity; bees that consumed sunflower pollen had 10-fold fewer *Crithidia* cells than bees that consumed buckwheat pollen (least squares means 4.3 cells µL^-1^ for sunflower-fed bees and 40.0 cells µL^-1^ for buckwheat-fed bees, pollen treatment: *χ^2^*_(1)_ = 21.5, *P* < 0.001).

Neither pollen diet nor *Crithidia* infection affected nectar consumption (pollen diet: *χ^2^*_(1)_ = 0.0004, *P* = 0.98; infection: *χ^2^*_(1)_ = 1.63, *P* = 0.20). However, there was a significant increase in nectar consumption over the course of the experiment (*χ^2^*_(1)_ = 21.51, *P* < 0.001). The consumption of sunflower pollen was stable over the course of the experiment, whereas consumption of buckwheat pollen declined over time (pollen x time interaction: *χ^2^*_(1)_ = 38.92, *P* < 0.001; Fig. S3). Averaged across all time points, sunflower pollen consumption was 2.3 times greater than buckwheat pollen (*χ^2^*_(1)_ = 66.67, *P* < 0.001; Mean ± SE g pollen, sunflower: 0.0607 g ± 0.0016 g, buckwheat: 0.026 g ± 0.0012 g; Fig. S3). *Crithidia* infection did not affect pollen usage and consumption (*χ^2^*_(1)_ = 1.89, *P* = 0.17).

Microcolonies in the sunflower pollen treatment laid eggs significantly more quickly than microcolonies fed buckwheat pollen, and were more than twice as likely to have laid eggs at any given time (hazard ratio = 2.13, *Z* = 2.85, *P* = 0.004). Similarly, microcolonies in the sunflower treatment produced significantly more larvae, and had heavier larvae (number of larvae: *χ^2^*_(1)_ = 8.37, *P* = 0.004; larval mass: *χ^2^*_(1)_ = 11.44, *P* < 0.001; Fig. 2). Ten microcolonies that were provided sunflower pollen produced pupae or adults, whereas none of the microcolonies that were provided buckwheat pollen produced pupae or adults (pollen treatment *χ^2^*_(1)_ = 7.89, *P* = 0.005). *Crithidia* infection did not affect time to egg laying (hazard ratio = 0.78, *Z* = -1.01, *P* = 0.31), number of larvae produced (*χ^2^*_(1)_ = 0.74, *P* = 0.39), larval mass (*χ^2^*_(1)_ = 0.017, *P* = 0. 0.89), or pupae production (*χ^2^*_(1)_ = 0.15, *P* = 0.69).

Both *Crithidia* infection and sunflower consumption marginally increased mortality rate (infection: *χ^2^*_(1)_ = 3.21, *P* = 0.07, pollen diet: see main text). Bees that consumed sunflower pollen were 31% more likely to die than those that consumed buckwheat pollen (death hazard ratio = 1.31 ± 0.165 SE; Fig. S4). Moreover, infected bees were 33% more likely to die at any given point than uninfected bees (death hazard ratio = 1.33 ± 0.161 SE; Fig. S4).

**3. Effects of pollen diet on *Nosema* in honey bees**. Pollen diet and time had a significant effect on *Nosema* infection intensity in honey bees (pollen treatment x time interaction: *χ^2^*_(2)_ = 48.2, *P* < 0.001). At each time point, sunflower-fed bees had on average fewer *Nosema* spores than buckwheat-fed bees (*Z* < -3.06, *P* < 0.01 for both time points), but more than bees fed no pollen (*Z* > 3.51, *P* < 0.01, Fig. 3). Pairwise comparisons indicate significantly higher mortality on both the sunflower pollen and no pollen diets relative to the buckwheat pollen diet (sunflower: see main text; no pollen: *Z* = 4.62, *P* < 0.001; Fig. S5). There was no significant difference in mortality between the sunflower and no pollen diets (*Z* = 0.75, *P* = 0.73).

**4. Effect of sunflower plantings on *Crithidia* in bumble bees at the farm scale.** Collection date was a significant covariate in the *Crithidia* infection intensity model (*χ^2^*_(1)_ = 14.46, *P* < 0.001). Sunflower area tended to reduce the probability of *Crithidia* infection (*χ^2^*_(1)_ = 2.92, *P* = 0.087), with increasing sunflower area associated with reduced probability of *Crithidia* infection.

**Supplementary Methods - statistical analyses:**

**1. Effects of pollen diet on *Crithidia* in bumble bees.**

*Effect of diet post-infection.* Bee size and inoculation date were not statistically significant (*P* > 0.2 for both) and so were removed from the model. None of the experimental bees died before their dissection date, and so a survival analysis was not conducted.

*Consistency with a different pathogen strain.* All bees were inoculated on the same day, so inoculation date was not included as a random effect in this analysis. Only two bees died before their dissection date, and so a survival analysis was not conducted.

*Consistency using two sources of sunflower pollen.* Bee size was not statistically significant (*P* = 0.95), and so was dropped from the model. A survival analysis was not conducted because only 10 bees died before their dissection date.

**2. Costs and benefits of sunflower pollen on bee health, reproduction and *Crithidia***. We excluded two microcolonies from the ‘uninfected’ treatment because one or more workers in the microcolony were infected with *Crithidia* upon dissection, and two microcolonies whose workers died within one week of inoculation. Significance of predictor variables was assessed with chi-squared tests (likelihood ratio tests for models fit by glmmTMB models, and Wald tests for all other models via the Anova function from package “car”^1^).

To model pollen and nectar consumption, a linear mixed effects model was employed using function lme in “lme4”^2^ with maximum likelihood parameter estimation. This was a repeated measures analysis including infection treatment, pollen diet, and days elapsed since inoculation as predictor variables. Two extreme daily consumption outliers were removed from the analyses. Survival and time to egg laying were analyzed with a Cox Proportional Hazards mixed-effects model fit using “coxme”^3^ with a maximum likelihood parameter estimation. Response variables for survival and egg laying were hazard rates, which incorporated both the event (survival/death, or eggs/no eggs) and time to the event. Egg laying occurred at the level of the microcolony, while death hazard rates were analyzed for each bee in the microcolony with bee nested inside microcolony. *Crithidia* infection intensity (cell counts) was analyzed for infected treatment colonies only, using a negative binomial error distribution and a log link function with Laplace approximation to likelihood by package “glmmTMB”^4^. Number of pupae/adults produced was low overall (only 11 out of 76 microcolonies produced any pupae or adults), and thus was recorded as a binary variable, analyzed with binomial error distribution in package "blme”^5^ with function bglmer, which uses Bayesian estimation to account for complete separation (i.e., fitted probabilities of zero or one in some treatments). Frequentist models for analysis of pupae failed to converge. This analysis only included blocks 1 and 2, as blocks 3 and 4 produced only one pupa and no adults. The number of larvae produced was analyzed with a negative binomial distribution and larval mass with a gamma distribution, each with Laplace estimation within package “glmmTMB”^4^. The number of eggs produced was analyzed with a Poisson error distribution and Gauss-Hermite quadrature estimation in package “lme4”^2^.

**3. Effects of pollen diet on *Nosema* in honey bees.** Significance of predictors was tested with likelihood ratio chi-squared tests via the drop1() function in R. Post-hoc pairwise comparisons were made with the “lsmeans” package^6^; the Tukey method was used to adjust p-values for multiple comparisons. The no-pollen negative control had significantly lower *Nosema* infection than either of the pollen diets at both days 10 and 15 (*Z* > 3.05, *P* < 0.01), as predicted, suggesting that the bees were consuming the pollen diets in the other treatments. Analysis of Deviance (Type II Wald chi-squared tests) was used to calculate the p-value. Pairwise comparisons between pollen diets were made using general linear hypothesis testing with single-step p-value corrections for multiple comparisons^6,7^.

**References**

1. Fox, J. & Weisburg, S. *An {R} Companion to Applied Regression*. (Sage, 2011).

2. Bates, D. M., Maechler, M., Bolker, B. & Walker, S. Fitting Linear Mixed-Effects Models Using lme4. *J. Stat. Softw.* **67,** 1–48 (2015).

3. Therneau, T. M. *coxme: Mixed Effects Cox Models. R package version 2.2-5*. (2015).

4. Magnusson, A. *et al.* glmmTMB: Generalized Linear Mixed Models using Template Model Builder. R package version 0.1.1. (2017).

5. Chung, Y., Rabe-Hesketh, S., Dorie, V., Gelman, A. & Liu, J. A nondegenerate penalized likelihood estimator for variance parameters in multilevel models. *Psychometrika* **78,** 685–709 (2013).

6. Lenth, R. *lsmeans: Least-Squares Means. R package version 2.20-23.* (2015).

7. Hothorn, T., Bretz, F. & Westfall, P. Simultaneous Inference in General Parametric Models. *Biom. J.* **50,** 346–363 (2008).

**Supporting Figures and Tables**

**Fig. S1.** Proportion of *Bombus impatiens* workers infected with *Crithidia* surviving over time for each of four pollen diets. “Buck” refers to buckwheat, *Fagopyrum cymosum*, “Rape” to *Brassica campestris*, “Mix” to a pollen diet composed of equal weights of the three monofloral pollens, and “Sun” to sunflower, *Helianthus annuus*. Time was measured in days (d).

**Fig. S2**. Effect of pollen diet on infection using individual *B. impatiens* workers that were inoculated with *Crithidia* sourced from J.C. Raulston Arboretum (35.793916 N, -78.698025 W), North Carolina, USA, and then fed either sunflower or buckwheat pollen. Bars and error bars indicate negative binomial model means and standard errors back-transformed (i.e., exponentiated) from the scale of the linear predictor. Different letters above each bar indicate significant differences.

**Fig. S3.** *Bombus impatiens* microcolony pollen consumption over time (days since inoculation) in µg bee^-1^ h^-1^ corrected for evaporation for buckwheat (solid line) and sunflower pollen (dashed line) treatments. Trend lines are overlaid over raw data; grey bands indicate ± one standard error.

**Fig. S4.** Proportion of *Bombus impatiens* workers in microcolonies surviving over time for each of four treatment combinations. Bees were either infected or uninfected with *Crithidia,* and fed sunflower pollen or buckwheat pollen. Time was measured in days (d).

**Fig. S5.** Proportion of individual honey bee workers infected with *Nosema* surviving over time for each of three pollen diets. Bees were provided sunflower pollen, buckwheat pollen or no pollen as a control. Time is measured in days.

**Supplementary Table 1.** Pollen diets were screened for 213 pesticides and other agrochemicals (Agricultural Marketing Services’ National Science Laboratories, United States Department of Agriculture, Gastonia, NC USA). We only report the chemicals detected in at least one of the pollen treatments and their concentrations in parts per billion (ppb). If concentrations were below detection limit, we report the limit of detection (LOD, in ppb) and list that the chemical was detected at trace concentrations. Not detected is listed as N.D. The highest pesticide concentrations were in the wildflower mix pollen, which also had relatively high *Crithidia* loads. Thus, pesticide contaminants do not explain the strong effect of sunflower pollen reducing *Crithidia* infection.
